# Association between the ratio of aspartate aminotransferase to alanine aminotransferase and risk of diabetes and the mediating effect of BMI: a comparative analysis in Chinese and Japanese populations

**DOI:** 10.3389/fendo.2025.1704211

**Published:** 2026-01-20

**Authors:** Mengyao Gu, Nan Niu, Yuheng Liao, Haofei Hu, Dayong Gu

**Affiliations:** 1 Department of Laboratory Medicine, The First Affiliated Hospital of Shenzhen University, Shenzhen, Guangdong, China; 2 Department of Laboratory Medicine, Shenzhen Second People’s Hospital, Shenzhen, Guangdong, China; 3 Department of Nephrology, The First Affiliated Hospital of Shenzhen University, Shenzhen, Guangdong, China; 4 Department of Nephrology, Shenzhen Second People’s Hospital, Shenzhen, Guangdong, China; 5 Department of Laboratory Medicine, Shenzhen Key Laboratory of Medical Laboratory and Molecular Diagnostics, The First Affiliated Hospital of Shenzhen University, Shenzhen Second People’s Hospital, Medical Innovation Technology Transformation Center of Shenzhen Second People’s Hospital, Shenzhen University, Shenzhen, China

**Keywords:** alanine aminotransferase, aspartate aminotransferase, body mass index, diabetes, East Asian populations, mediation analysis, population comparison, threshold effect

## Abstract

**Objective:**

Despite the AST/ALT ratio emerging as a promising diabetes predictor, current research lacks comprehensive understanding of population-specific patterns and underlying mechanisms. This study aims to investigate the AST/ALT-diabetes risk relationship in Chinese and Japanese populations, specifically examining non-linear relationships, population-specific thresholds, and BMI’s potential mediating role to address existing knowledge gaps.

**Methods:**

We performed a retrospective cohort analysis using data from the China Rich Healthcare Group (n=84,281) and Japanese NAGALA database (n=15,291). AST/ALT ratio was calculated by dividing AST concentration by ALT concentration. Diabetes was defined by fasting plasma glucose ≥7.00mmol/L or self-reported diagnosis. We employed Cox proportional hazards models, including restricted cubic splines to explore non-linear relationships, two-piecewise regression for identifying population-specific thresholds, and mediation analysis to quantify BMI’s mediating effect on the AST/ALT-diabetes risk association.

**Results:**

In a cohort of 99,572 participants with 1,403 new diabetes cases, AST/ALT showed a significant negative association with diabetes risk across Chinese and Japanese populations (HR per unit increase: Chinese: 0.417, 95% CI: 0.341-0.510; Japanese: 0.631, 95% CI: 0.416-0.956). Country-specific variations were evident, with distinct non-linear relationships: Chinese participants exhibited risk reduction until AST/ALT reached 0.912, while Japanese participants showed an inflection point at 0.882. BMI’s mediating effect differed markedly between populations, with a higher proportion in Japanese participants (32.22%), suggesting nuanced pathophysiological mechanisms underlying the AST/ALT-diabetes relationship.

**Conclusions:**

Our study unveils significant ethnic variations in the AST/ALT-diabetes interaction, highlighting population-specific diagnostic thresholds and mediation mechanisms. These insights advocate for customized screening approaches and suggest that AST/ALT and BMI-targeted interventions may yield differential outcomes across East Asian populations, emphasizing the critical need for precision-based medical strategies.

## Introduction

Diabetes mellitus represents a complex metabolic syndrome characterized by persistent elevated blood glucose levels, predominantly categorized into two primary variants: insulin-dependent Type 1 diabetes (T1D) and insulin-resistant Type 2 diabetes (T2D). The global impact of this metabolic disorder extends beyond individual health, encompassing substantial clinical complications and significant socioeconomic challenges worldwide. The progression of diabetes is often accompanied by various complications such as diabetic peripheral neuropathy, and diabetic nephropathy, which seriously affect patients’ quality of life (1, 2). The International Diabetes Federation(2025) reported that there are around 589 million adults (20-79 years) globally with diabetes, representing 11.1% of the adult population, and it is projected that the number will reach 853 million by 2050. The global economic burden has exceeded one trillion US dollars annually (3, 4). Although obesity rates are lower than Western populations, China and Japan have reached alarming levels of diabetes prevalence. In China, approximately 140.9 million adults (13% prevalence) have diabetes. In Japan, an estimated 11 million cases (11.8% prevalence) have been reported. In view of the adverse impact of diabetes on both patients and society, timely diabetes identification and management are especially urgent, particularly in China and Japan. One approach is to seek potential risk factors for diabetes.

The liver plays an important role in the pathogenesis of metabolic syndrome as an essential organ in maintaining glucose homeostasis and modulating insulin resistance (5). Diabetes often coexists with nonalcoholic fatty liver disease (NAFLD) in individuals, which is commonly recognized as a metabolic syndrome-associated hepatic lipid accumulation disorder (6, 7). Epidemiological research has reported that more than half of patients with diabetes will suffer from NAFLD (8), and individuals diagnosed with NAFLD have a significantly increased risk of developing diabetes, approximately double the risk compared to healthy individuals (9).

In individuals with NAFLD, circulating alanine aminotransferase (ALT) and aspartate aminotransferase (AST) often change significantly. These enzymes are representative transaminases used to assess liver health, especially reflecting the accumulation of fat in the liver (10–12). ALT acts as a biomarker for liver damage, and it is involved in the process by which L-alanine and 2-oxoglutarate are transformed to pyruvate and L-glutamate in liver cells for cellular energy production. This process further involves the subsequent conversion of pyruvate to lactate, during which NADH is oxidized to NAD (13). AST, a prototypical pyridoxal 5’-phosphate (PLP) dependent enzyme, has the ability to catalyze the reversible interconversion of L-aspartate and α-ketoglutarate with oxaloacetate and L-glutamate via a ping-pong catalytic cycle (14), AST is associated with various conditions, including alcoholic liver disease, viral hepatitis, cirrhosis, acute myocardial infarction, cholestatic syndromes, or skeletal muscle trauma (15).

The early longitudinal investigations examining the relationship between NAFLD biomarkers and diabetes risk were published in 1998 (16). Since then, an increasing number of studies have focused on the associations between AST and ALT, and diabetes (17–21). Framingham investigators revealed that the AST/ALT ratio offers enhanced diagnostic sensitivity for identifying steatotic liver disease beyond single enzyme evaluation (22), thereafter the AST/ALT ratio garnered particular attention. Recently, a growing body of research has elucidated its predictive capacities for other diseases including peripheral arterial disease, metabolic syndrome, and chronic kidney disease, in addition to NAFLD (23–26). Meanwhile, The lower ALT/AST ratio emerges as a critical biomarker signaling potential insulin resistance and its multifaceted metabolic sequelae (11, 27–29). Subsequent research has demonstrated that a diminished AST/ALT ratio emerges as a highly sensitive predictive marker for diabetes onset within Asian demographic cohorts (30–32).

In China, research on the AST/ALT ratio-diabetes relationship still remains limited. Cross-sectional studies consider the ALT/AST ratio as one of the best predictors for insulin resistance, with an area under the receiver operating characteristic (ROC) curve (AUC) of 0.66 (95% confidence interval (CI), 0.64-0.68) in subjects without central obesity and 0.68 (0.66-0.70) in subjects with central obesity (29). To address the limitations of the cross-sectional nature, Xie et al. conducted a cohort analysis, and demonstrated that an increase in the AST/ALT ratio was associated with a reduced risk of diabetes (HR: 0.56, 95% CI: 0.37-0.85, P = 0.006) after further adjustment. Investigative findings uncovered a nuanced correlation wherein the AST/ALT ratio below 1.18 is associated with a dramatic increase in diabetes risk. In contrast, values surpassing this threshold exhibit minimal independent impact on diabetes progression (33). New insights into prediabetes indicate that each unit increase in the AST/ALT ratio reduces the risk of prediabetes by 21%, with a non-linear association also observed (34).

In Japanese populations, emerging evidence supports the predictive value of the AST/ALT ratio for diabetes. Cross-sectional studies point to a positive relationship between the ALT/AST ratio and insulin resistance through linear correlation analysis in specific populations (11, 35). Chen et al. conducted a retrospective study and discovered that the AST/ALT ratio is an independent predictor of the incidence of T2DM in the fully adjusted model (HR: 0.617, 95% CI: 0.405-0.938, P = 0.0239). A non-linear relationship and saturation effect were further found, below the inflection point of 0.882 in the AST/ALT ratio, analysis revealed a significant inverse correlation between the AST/ALT ratio and T2D incidence, demonstrating a pronounced negative association (HR: 0.287). Beyond this critical inflection point, a saturation effect was observed (32). Additionally, a cut-off value of 0.875 for the AST/ALT ratio was assessed for predicting 10-year incidence of T2D through ROC and AUC analyses in another cohort study (31).

Although there is rising interest in using the AST/ALT ratio as a potential predictor for diabetes in Chinese and Japanese populations, the existing research has limitations. Cross-sectional research designs make it impossible to draw causal inferences and establish temporal relationships. In addition, considerable heterogeneity exists in study populations, with research focusing on either general populations or specific high-risk groups, limiting the generalizability of the results. Moreover, methodological inconsistencies-–including variations in follow-up times, definition of diabetes, and confounding factor adjustments-–complicate the interpretation and comparison of results across studies.

The Chinese and Japanese populations possess distinct dietary habits, and environmental exposures, which offer a unique vantage-point to explore potential ethnic disparities in the relationship between the AST/ALT ratio and diabetes. Despite these differences, these two populations share some commonalities. For instance, compared to Western populations, they generally have lower obesity rates. However, they display significant variations in the prevalence of diabetes and the profiles of associated risk factors (36). Delving into these differences can offer valuable perspectives on the underlying mechanisms, and further contribute to the refinement of risk stratification methods, enabling more targeted and effective prevention and management strategies.

Obesity is related to hepatic fat accumulation, which could result in elevated AST level and ALT level. This liver dysfunction may further impair insulin sensitivity, thereby increasing the risk of diabetes (37). BMI, a widely used measure to assess obesity, may serve as an important mediator in the relationship between AST/ALT and diabetes. Given the nuanced differences in body composition and metabolic profiles between Chinese and Japanese populations, we hypothesize that the mediating effect of BMI on this relationship may vary between these two cohorts.

Therefore, the present study aims to address these limitations through a retrospective cohort analysis of the AST/ALT ratio-diabetes relationship in both Chinese and Japanese populations. This comparative approach represents a significant innovation, allowing identification of population-specific threshold values and risk patterns. By simultaneously analyzing data from two distinct East Asian cohorts with similar genetic backgrounds but different lifestyle exposures, we can explore both the consistency of the AST/ALT-diabetes relationship across populations and potential variations in its manifestation. Our study incorporates comprehensive assessments of linear and non-linear relationships, explores potential effect modifications by demographic and clinical factors, and evaluates the predictive performance of the ASL/ALT ratio for diabetes incidence risk. We further conduct mediation analysis to explore whether BMI plays a mediating role in the relationship between AST/ALT levels and diabetes.

In summary, we hypothesize that the inverse association between the AST/ALT ratio and diabetes will be consistent across both Chinese and Japanese cohorts, supporting its role as a robust pan-Asian biomarker, and BMI will mediate this association, which may vary between these two cohorts.

## Methods

### Study design and data sources

This retrospective longitudinal investigation examined the intricate relationship between the AST/ALT ratio and the incidence of diabetes across two geographically distinct East Asian population cohorts. It leveraged medical datasets from Chinese and Japanese medical research repositories: the China Rich Healthcare Group database (38) and the Japanese NAGALA (NAFLD in the Gifu Area, Longitudinal Analysis) database (39).

The study’s Chinese cohort data originated from a health examination database meticulously compiled by the Rich Healthcare Group. This expansive dataset encompassed medical records from 32 clinical sites across 11 prominent Chinese urban centers, including metropolises such as Beijing, Shanghai, Guangzhou, and Shenzhen. Data collection occurred between 2010 and 2016, employing a systematic, consecutive, and non-discriminatory participant recruitment strategy, designed to minimize potential selection bias and optimize data representativeness and scientific integrity (40). The raw data were obtained from the Dryad open-access repository, specifically from a dataset provided by Chen, Ying et al. (2018), titled “Association of body mass index and age with incident diabetes in Chinese adults: a population-based cohort study” (40).

The Japanese research cohort was selected from the NAGALA database (NAFLD in the Gifu Area, Longitudinal Analysis), a medical records collection developed by Murakami Memorial Hospital. The study conducted between 2004 and 2015, employed a careful recruitment strategy that prioritized consecutive and non-selective participant inclusion, to minimize sampling bias and enhance data reliability. The original dataset is freely accessible through the Dryad open-access repository, and was initially published by Okamura, et al. (2019) in their study on ectopic fat accumulation and type 2 diabetes risk (41).

In accordance with Dryad’s open-access research protocol, the database is disseminated to support secondary analyses while carefully preserving the intellectual property rights of the original researchers and promoting transparent scientific knowledge exchange.

### Study population

#### Chinese cohort

**Figure 1:**
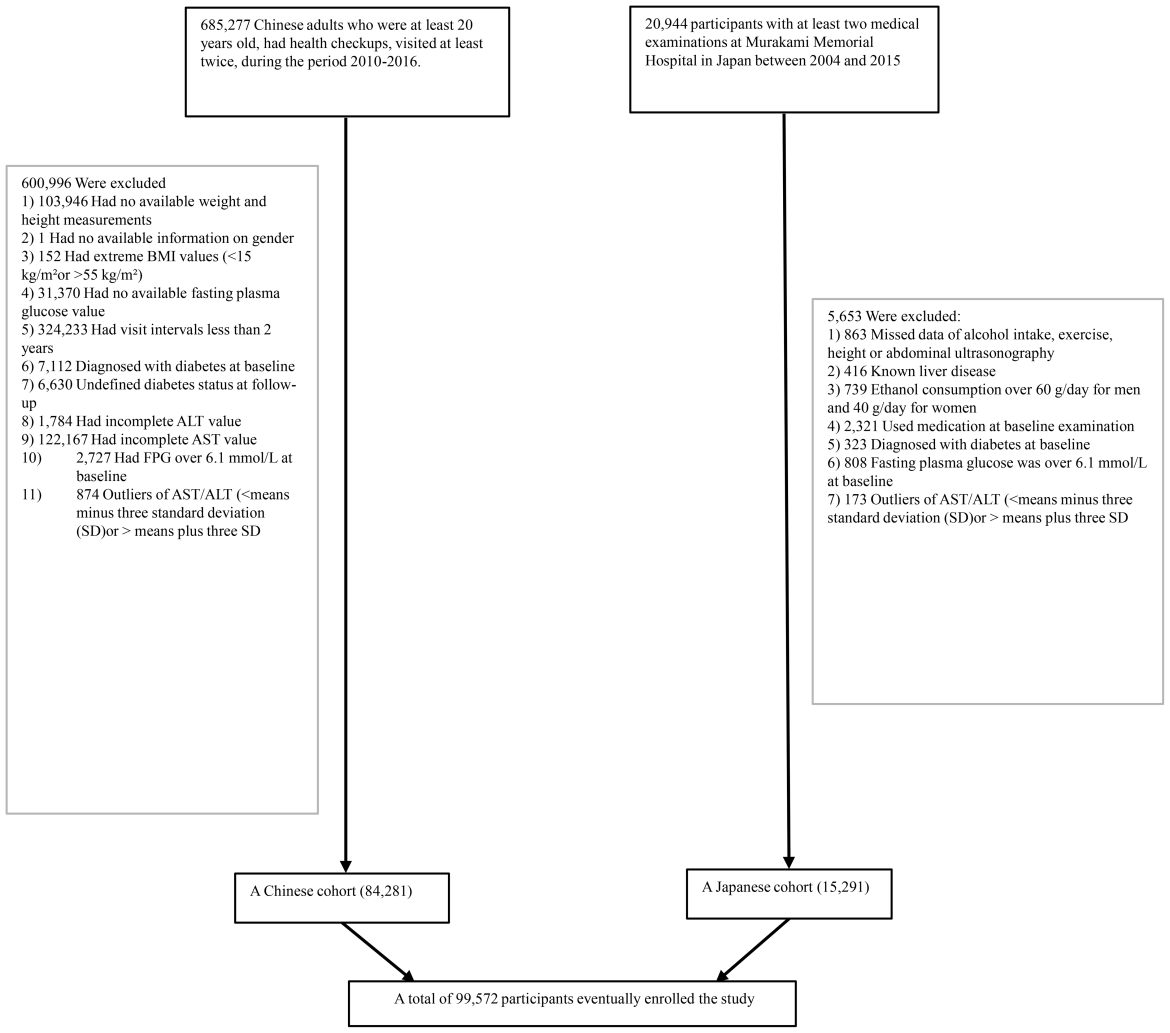
The flowchart of the study.

From an initial comprehensive health screening involving 685,277 participants, the systematic screening process eliminated participants based on multiple precise criteria, including incomplete baseline demographic and physiological data (n=135,317), inadequate longitudinal monitoring duration (<2 years) (n=324,233), BMI values outside the physiological range (<15 kg/m^2^ or >55 kg/m^2^) (n=152), unconfirmed diabetes diagnosis status (n=6,630), pre-existing diabetes at baseline (n=7,112), missing or incomplete AST or ALT data (n=123,951), elevated baseline fasting plasma glucose levels (FPG > 6.l mmol/L) (n=2,727), and AST/ALT outliers defined as values below the mean minus three standard deviations (SD) or above the mean plus three SD (n=874). This selection protocol was strategically designed to minimize bias. Through this data refinement process, we systematically narrowed the initial expansive population, ultimately retaining 84,281 Chinese participants who met our stringent scientific standards. (see **Figure 1** for detailed flowchart).

#### Japanese cohort

The Japanese research population initially comprised 20,944 individuals, who underwent health examinations. We applied the following exclusion criteria: participants including those with confirmed diabetes diagnoses (n=323) or elevated baseline FPG levels (FPG>6.1 mmol/L) (n=808), individuals with documented hepatic pathologies such as hepatitis B or C infections (n=416), participants receiving pharmaceutical treatments at baseline (n=2,321), and those demonstrating alcohol consumption exceeding gender-specific thresholds (more than 40 grams per day for women and more than 60 grams per day for men) (n=739). A further refined selection process excluded participants with incomplete critical covariate, data due to missing abdominal ultrasonographic data, exercise patterns, alcohol intake, and essential laboratory variables (n=863), Separately, a statistical normalization of biochemical indicators was conducted by identifying and removing outliers beyond three standard deviations in serum AST and ALT levels (n=173). This exclusion strategy systematically distilled the initial expansive population, ultimately retaining 15,291 valid participants from the Japanese cohort.

By integrating this dataset with the previously analyzed Chinese cohort, the study consolidated a comprehensive total of 99,572 participants (see **Figure 1** for detailed flowchart).

### Ethics statement

Ethical approval for the Chinese cohort research was formally granted by the Ethics Committee of the Rich Healthcare Group, and the Japanese cohort study received ethical approval from the Ethics Committee of Murakami Memorial Hospital. Both studies adhered to the Declaration of Helsinki, and informed consent was waived due to the retrospective nature of the study (42). Since the retrospective and secondary data sources used in this study were completely de-identified, additional ethical approval was not required.

### Variables

#### The AST/ALT ratio

The primary exposure variable was established as the AST/ALT ratio, mathematically derived by dividing the AST concentration (U/L) by the ALT concentration (U/L). Both enzymatic markers were quantified using an automated biochemical analyzer following a standardized fasting period exceeding 8 hours.

#### Outcome Measures

Diabetes was the focal dichotomous outcome variable, systematically coded with binary classification (0: absence, 1: presence). Participant monitoring ended at the earliest of diabetes diagnosis or the final study visit. Incident diabetes diagnostic criteria encompassed two complementary parameters: objectively measured fasting plasma glucose ≥7.00mmol/L and/or self-reported diabetes diagnosis by participants during the investigative follow-up interval (43).

### Data collection and Covariates

The investigative framework encompassed comprehensive data collection from Chinese and Japanese cohorts, systematically aggregating a multidimensional dataset featuring critical health parameters. The integrated variable profile included extensive demographic descriptors, metabolic indicators, cardiovascular measurements, lipid profile components, hepatic enzymatic markers, and lifestyle variables.

Tobacco exposure was dichotomized into two distinct groups “ever smokers and never smokers” based on lifetime smoking history, this classification clearly separated individuals with a history of tobacco consumption from those maintaining a lifelong non-smoking status. Similarly, alcohol intake was categorized as “ever drinkers” or “never drinkers”.

The comprehensive physiological assessment was conducted by professionally trained personnel following stringent standardized procedures. Anthropometric evaluations were performed under controlled conditions, with participants minimally attired in lightweight clothing and standing barefoot to ensure precise height and weight measurements. BMI was calculated using the standard formula: weight in kilograms divided by height in meters squared. Blood pressure was measured with calibrated sphygmomanometers after participants had rested for 5 to 10 minutes under standardized conditions to stabilize physiological parameters. Biological sample collection followed strict pre-analytical protocols, requiring participants to fast overnight for at least 10 hours. Subsequent biochemical analyses were carried out in specialized laboratories using automated analytical systems.

Covariate identification was based on a structured methodological framework that integrated current clinical knowledge with extensive literature-based evidence (44, 45). The variables included were age, gender, BMI, smoking status, drinking status, SBP, DBP, FPG, TC, TG, LDL-c, HDL-c, ALT, and AST. In Japanese populations LDL-C (mg/dl) was calculated using the formula: [TC(mg/dl)-HDL(mg/dl)]*90% minus TG(mg/dl)*10% (46).

### Missing data processing

The Chinese dataset contained different proportions of missing values across different variables: SBP (n=10, 0.012%), DBP (n=11, 0.013%), TC (n=1078, 1.279%), TG (n=1673, 1.985%), HDL-c (n=36898, 43.780%) and LDL-c (n=36200, 42.952%), smoking status (n=63302, 75.108%), drinking status (n=63302, 75.108%). Meanwhile, the Japanese dataset contained missing data: HDL-c (n=11, 0.072%) and LDL-c (n=11, 0.072%). To address potential bias and optimize data utilization, we conducted five multiple imputation by chained equations on these two datasets (Chinese and Japanese) (47). The variables used for imputation included BMI, age, gender, FPG, DBP, SBP, ALT, AST, TC, HDL-c, TG, LDL-c, and smoking and drinking status. The missing data were analyzed based on the assumption that the missing data were missing at random (MAR) (48).

### Statistical analysis

Baseline characteristics were analyzed across AST/ALT quartiles (Q1:<0.8666; Q2:0.8666-1.1481; Q3:1.1481-1.4749; Q4: ≥1.4749) using appropriate statistical methods. Continuous variables underwent comprehensive statistical characterization, with normally distributed data represented by mean and standard deviation, while skewed distributions were described using median and interquartile range statistics. Categorical variables were systematically documented through frequency and percentage analyses. Statistical comparisons used appropriate analytical techniques, utilizing one-way ANOVA for parametric continuous variables, the Kruskal-Wallis test for non-parametric distributions, and chi-square tests for categorical data. The survival analysis component implemented the Kaplan-Meier method, with the log-rank test applied to generate comparative analyses of cumulative hazard across distinct AST/ALT groupings. To aligns the time windows for the Chinese and Japanese cohorts, we have defined the survival status of participants in the Overall database, who experienced a diabetes event after 6 years of follow-up, as not having experienced the outcome event. For other participants, their survival status remains unadjusted. We have standardized the follow-up duration for the overall databases to 6 years, where follow-up times exceeding 6 years are categorized as 6 years, and those within 6 years retain their original follow-up times without adjustments.

To assess multicollinearity among covariates, researchers employed the variance inflation factor (VIF). This technique quantifies the interdependence between predictor variables by utilizing a computational method expressed as 1/(1-R²), where R² represents the coefficient of determination derived from a linear regression model (49). The analytical protocol involved a comprehensive evaluation where each predictor underwent regression testing against the remaining variables. When the VIF surpassed the threshold of 5, it led to exclusion from the multiple regression model (**Supplementary Table S1**).

To analyze the association between the AST/ALT ratio and diabetes, we conducted the following analytical steps:

Step 1: Univariate and multivariate cox proportional-hazards regression

Cox proportional hazards regression models were implemented to explore the association between AST/ALT and incident diabetes risk in the overall and specific populations. We constructed three progressively adjusted models:

Model 1: Unadjusted
Model 2: Adjusted for age, gender, BMI, SBP, DBP, smoking status, and drinking status
Model 3: Adjusted for age, gender, BMI, SBP, DBP, smoking status, and drinking status, FPG, TG, HDL-C, LDL-C.

Hazard ratios (HRs) and their corresponding 95% confidence intervals (CIs) were computed in the statistical analysis. These HRs were calculated across different AST/ALT quartiles, with the lowest quartile serving as the reference group. Additionally, a separate analysis examined AST/ALT as a continuous variable. The HR per one unit increase in AST/ALT was evaluated to provide a comprehensive assessment of potential risk relationships.

Furthermore, the covariate selection process drew upon existing scholarly research and comprehensive collinearity evaluations. Following examination, total cholesterol (TC) was omitted from the multivariate analysis due to observed collinearity (**Supplementary Table S1**). Methodological validation of proportional hazards assumptions was conducted using Schoenfeld residuals and log-minus-log diagnostic plots. The analytical framework was designed to systematically assess variations in effect estimate under different adjustment scenarios.

Step 2: Nonlinearity and two-piece regression analysis

The research explored the potential nonlinear dynamics between AST/ALT and diabetes risk through advanced statistical modeling. A Cox proportional hazards regression approach with cubic spline functions and smooth curve fitting (utilizing the penalized spline method) was deployed to examine complex relationship patterns. Upon detecting nonlinearity, a recursive algorithm precisely identified the critical inflection point. Subsequent a two-piece Cox proportional hazards regression model was constructed, strategically segmenting the data around this pivotal point. To validate the analytical approach, a comparative sensitivity analysis was conducted, juxtaposing the standard Cox proportional hazards regression model against the two-piece model. The likelihood ratio test was rigorously applied to determine the most optimal statistical framework for elucidating the intricate AST/ALT-diabetes risk association.

Step 3: Subgroup analysis and sensitivity analysis

The analysis employed a stratified Cox proportional-hazards regression model to examine potential heterogeneity across diverse demographic and clinical subgroups. These stratifications encompassed multiple parameters including gender, BMI, age, SBP, DBP, alcohol consumption, smoking habits, and geographical variations. To facilitate nuanced comparative analysis, continuous variables were systematically transformed into categorical classifications using predefined clinical threshold values, enabling more structured and interpretable statistical assessment. The categories were as follows: age (0 to <20, 20 to <45, 45 to <60, ≥60 years), BMI (<18.5, ≥18.5 to <24, ≥24 to <28, ≥28 kg/m^2^), SBP (<140, ≥140mmHg), and DBP (<90, ≥90mmHg) (4, 45). Each stratification was adjusted for all other factors in addition to the stratification factor itself (gender, age, BMI, SBP, DBP, FPG, TG, HDL-c, LDL-c, drinking and smoking status). Likelihood ratio test was conducted to compare models with and without interaction terms to assess potential interactions between variables.

To enhance the robustness of the study outcomes, comprehensive sensitivity analyses were implemented. The AST/ALT measurements were strategically reclassified into quartile-based classifications. A trend analysis was subsequently conducted to explore potential non-linear associations and cross-validate the findings originally derived from the continuous variable approach. This study, acknowledging the intricate interconnections between lifestyle variables, metabolic profiles, and diabetes development, implemented nuanced sensitivity analyses. These methodological refinements eliminated participant subgroups with predefined characteristics potentially confounding diabetes risk assessment: those with histories of smoking and drinking (50), individuals with obesity (BMI ≥28 kg/m^2^), and participants presenting with hypertriglyceridemia (TG ≥1.7 mmol/L) (51, 52).

Step 4: Predictive value of AST/ALT for diabetes risk

The discriminative capability of AST/ALT was quantitatively evaluated using the AUC with a fixed follow-up duration. The AUC metric ranges from 0.5, indicating no predictive power, to 1.0, representing perfect discrimination. The Youden index, calculated by the sum of sensitivity and specificity minus one, was employed to identify the optimal AST/ALT threshold for diabetes prediction. Furthermore, ethnicity-based stratification was conducted to assess the consistency of predictive performance across different population subgroups.

Step 5: Mediation analysis of BMI in the AST/ALT-diabetes association

To elucidate the mechanistic links between AST/ALT and diabetes risk, we conducted a comprehensive mediation analysis based on VanderWeele’s methodological framework (53, 54). This approach decomposed the total effect of AST/ALT on diabetes risk into two distinct pathways: a direct effect (AST/ALT→diabetes) and an indirect effect mediated through body mass index (AST/ALT→BMI→diabetes), thereby providing detailed insights into the potential physiological mechanisms underlying this complex relationship.

We applied Cox proportional hazards models combined with bootstrapping techniques involving 5,000 statistical replications to estimate HRs with 95% CIs. This method allowed precise estimation of direct, indirect, and total effects within the study’s analytical framework (54, 55). The mediation proportion, defined as the percentage of the total effect mediated through the BMI pathway, was calculated by dividing the natural indirect effect by the total effect (56). All mediation models were adjusted for potential confounders selected based on prior literature and clinical relevance, including age, gender, SBP, DBP, FPG, LDL-c, HDL-c, TG, smoking status, and drinking status. Analyses were performed for both the overall and ethnicity-specific populations.

### Statistical software

All statistical analyses were performed using R software (http://www.R-project.org, the R Foundation) and EmpowerStats software (X&Y Solutions, Inc; http://www.empowerstats.com). Statistical significance was defined as two-sided P < 0.05 to account for effects in both directions.

## Results

### Baseline characteristics of participants

The study cohort’s demographic profile, detailed in **Table 1**, revealed a participant population with a mean age of 42.04 ± 12.06 years, predominantly male (56.55%). The baseline AST/ALT ratio averaged 1.20 ± 0.44. Throughout a median follow-up period of 3.59 years, 1,403 participants (1.41%) developed diabetes.

**Table 1:** The baseline characteristics of participants.

AST/ALT	Q1 (<0.8666)	Q2 (0.8666-1.1481)	Q3 (1.1481-1.4749)	Q4 (≥1.4749)	P-value
N	24847	24910	24920	24895	
Age(years)	40.29 ± 9.74	42.95 ± 11.56	42.93 ± 12.73	41.97± 13.66	<0.001
BMI(kg/m^2^)	25.02 ± 3.30	23.35 ± 3.09	22.35 ± 2.96	21.48 ± 2.73	<0.001
SBP(mmHg)	122.19 ± 15.14	119.03 ± 15.97	117.04 ± 16.21	114.91 ± 16.42	<0.001
DBP(mmHg)	76.58 ± 10.78	74.24 ± 10.73	72.57 ± 10.50	71.16 ± 10.31	<0.001
FPG(mmol/L)	5.03 ± 0.54	4.96 ± 0.54	4.90 ± 0.54	4.83 ± 0.56	<0.001
TC(mmol/L)	4.94 ± 0.92	4.79 ± 0.89	4.68 ± 0.90	4.59 ± 0.88	<0.001
TG(mmol/L)	1.38 (0.91-2.04)	1.07 (0.72-1.60)	0.90 (0.63-1.32)	0.80 (0.60-1.14)	<0.001
HDL-c(mmol/L)	1.29 ± 0.30	1.37 ± 0.31	1.42 ± 0.31	1.45 ± 0.30	<0.001
LDL-c(mmol/L)	2.93 ± 0.73	2.80 ± 0.69	2.71 ± 0.68	2.63 ± 0.66	<0.001
ALT(U/L)	35.30 (27.00-49.30)	21.00 (17.00-26.00)	15.60 (13.00-18.60)	11.10 (9.60-13.30)	<0.001
AST(U/L)	25.00 (19.60-31.80)	21.00 (17.80-25.50)	20.00 (17.00-23.90)	20.00 (17.30-23.20)	<0.001
Gender, n(%)					<0.001
Female	3819 (15.37%)	8811 (35.37%)	13198 (52.96%)	17436 (70.04%)	
Male	21028 (84.63%)	16099 (64.63%)	11722 (47.04%)	7459 (29.96%)	
Smoking status, n(%)					<0.001
Non-smoker	15369 (61.85%)	17958 (72.09%)	20229 (81.18%)	22165 (89.03%)	
Smoker	9478 (38.15%)	6952 (27.91%)	4691 (18.82%)	2730 (10.97%)	
Drinking status, n(%)					<0.001
Non-drinker	18244 (73.43%)	19610 (78.72%)	20850 (83.67%)	22159 (89.01%)	
Drinker	6603 (26.57%)	5300 (21.28%)	4070 (16.33%)	2736 (10.99%)	
Country, n(%)					<0.001
China	19592 (78.85%)	19954 (80.10%)	21513 (86.33%)	23222 (93.28%)	
Japan	5255 (21.15%)	4956 (19.90%)	3407 (13.67%)	1673 (6.72%)	

Values are n (%), mean ± SD or medians (quartiles).

BMI, body mass index; SBP, systolic blood pressure; DBP, diastolic blood pressure; FPG, fasting plasma glucose; TC, total cholesterol;TG, triglyceride; HDL-c, high-density lipoprotein cholesterol; LDL-c, low-density lipid cholesterol; AST, aspartate aminotransferase; ALT, alanine aminotransferase.

Participants were stratified into subgroups using AST/ALT quartiles (<0.8666, 0.8666-1.1481, 1.1481-1.4749, ≥1.4749). Compared with the Q1(<0.8666) group, the other groups revealed statistically significant variations (p<0.001). The Q4 group exhibited marked reductions in the proportion of male, the prevalence of smoking and drinking, the proportion of Japanese participants, ALT, AST, TG, TC, LDL-c, FPG, SBP and DBP, and BMI. Conversely, HDL-c levels and the number of Chinese participants showed an opposite trend. All comparative metrics achieved statistical significance. **Supplementary Table S2** delineates the demographic and clinical distinctions between the Chinese (n=84,281) and Japanese (n=15,291) cohorts. Significant inter-population variations were observed: Japanese participants were older (43.72 vs. 41.73 years), had a higher proportion of females (45.18% vs. 43.14%), and notably higher prevalences of smoking (41.77% vs. 20.72%) and drinking (23.74% vs. 17.89%). Conversely, Chinese participants exhibited higher BMI (23.22 vs. 22.13 kg/m²), TG, AST, ALT, and blood pressure values. Japanese participants demonstrated higher levels of FPG, TC, LDL-c, and HDL-c. All comparative differences achieved statistical significance (p<0.001), underscoring the substantial metabolic and lifestyle disparities between the two populations.

**Figure 2:**
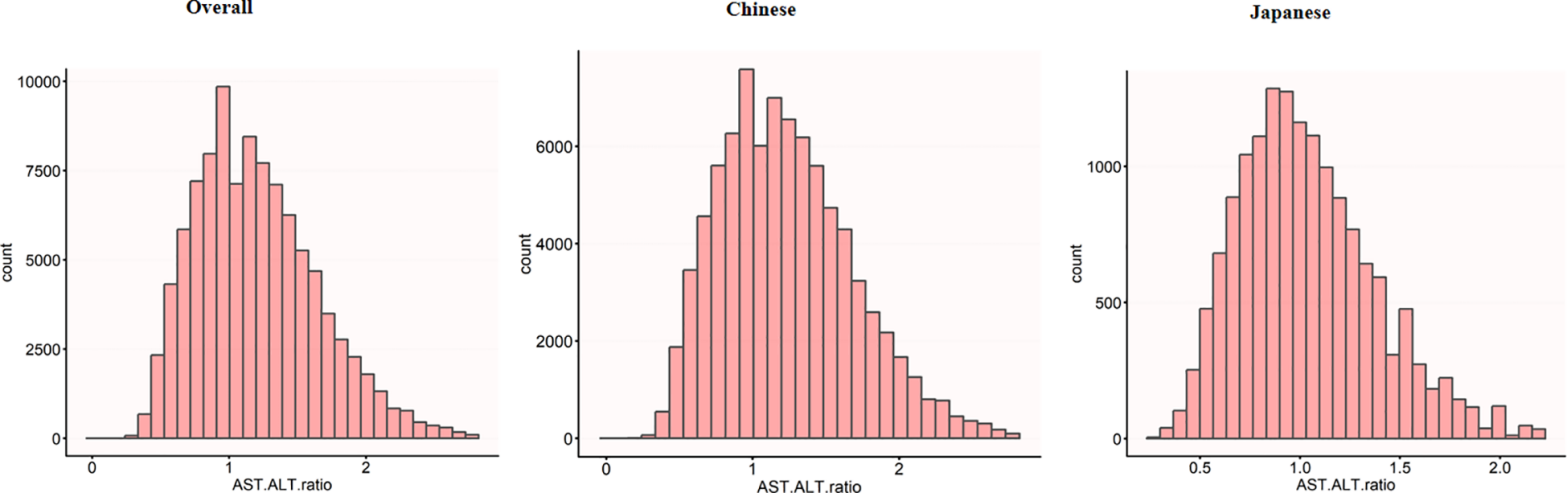
Distribution of AST/ALT across different populations. The histograms show the distribution of AST/ALT values in the overall study population (n=99,572), the Chinese subpopulation (n=84,281), and the Japanese subpopulation (n=15,291). The x-axis represents AST/ALT values, while the y-axis shows the count of individuals. The overall population showed a mean AST/ALT of 1.20 (SD 0.44), while the Chinese and Japanese populations had mean AST/ALT values of 1.23 (SD 0.45) and 1.04 (SD 0.34), respectively.

**Figure 2** presents a comprehensive visualization of AST to ALT ratio distribution across three population cohorts. The graphical representation features histograms that capture the normal distribution characteristics of AST to ALT ratios for the aggregate study population (panel A), Chinese participants (panel B), and Japanese participants (panel C).

**Figure 3:**
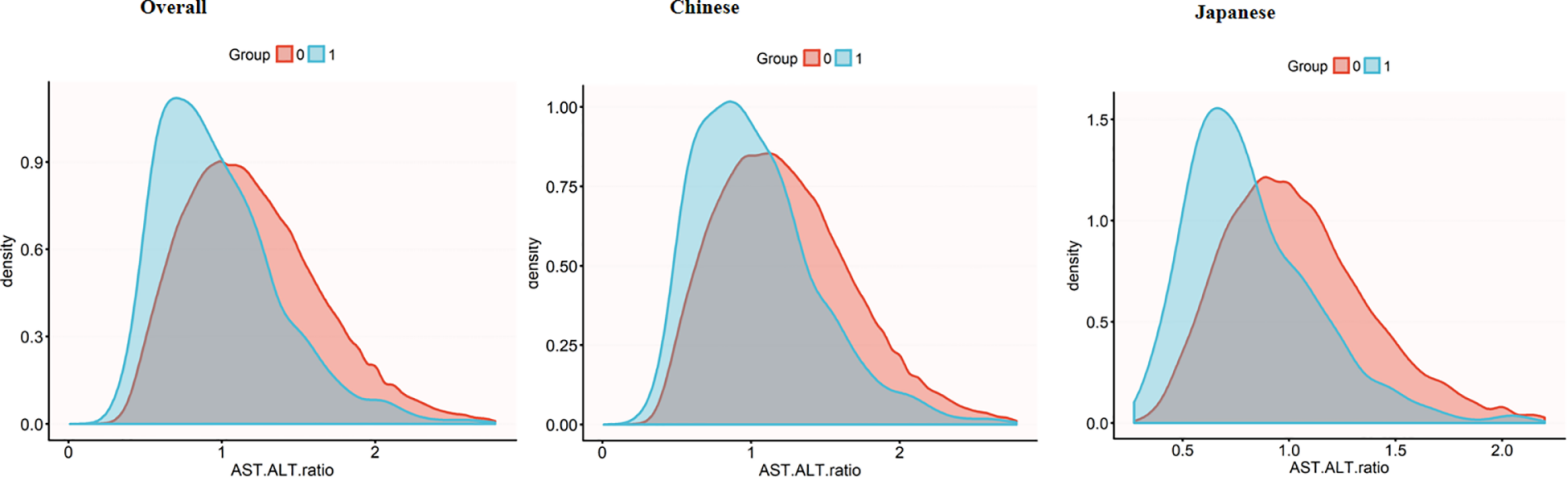
AST/ALT density distribution by diabetes status across populations. Density plots showing AST/ALT ratio distributions in the overall population (left), Chinese subgroup (middle), and Japanese subgroup (right). Diabetic groups (blue) consistently demonstrates lower AST/ALT ratios compared to non-diabetic groups (red), with this difference being more pronounced in the Japanese subgroup. AST, aspartate aminotransferase; ALT, alanine aminotransferase.

**Figure 3** reveals divergent AST to ALT ratio distribution patterns between diabetic and non-diabetic participants across populations. Diabetic participants demonstrate lower ratio peaks, whereas non-diabetic participants exhibit a distribution shifted to the right with a more pronounced extension in the right-tail of the distribution. Notably, the Japanese participants exhibit this phenomenon more clearly. In this subgroup, the distributional differences between diabetic and non-diabetic participants are more distinct, diabetic participants show a more significant rightward extension, thereby accentuating the variations in AST to ALT ratio within this population.

### The incidence rate of diabetes

**Figure 4:**
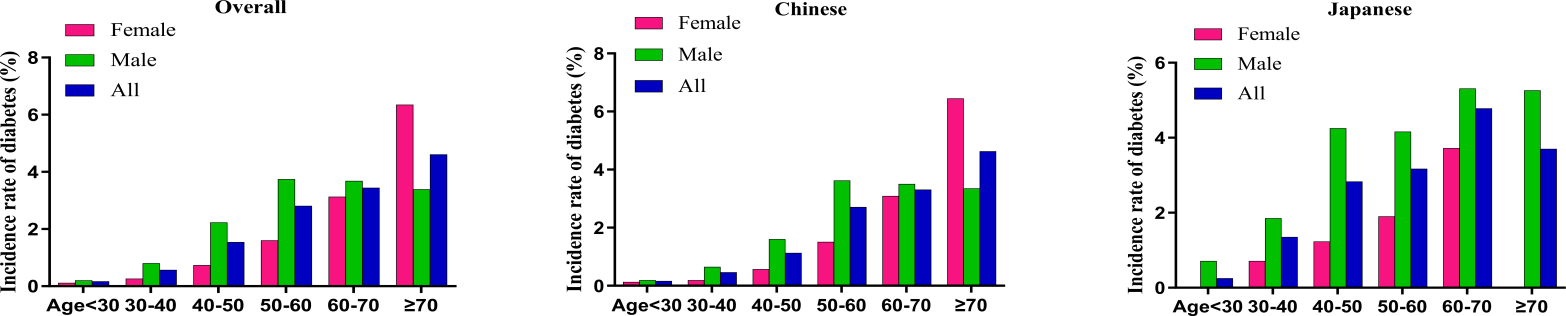
Age-specific incidence rates of diabetes stratified by sex and ethnicity The figure depicts the incidence rates of diabetes (%) across different age groups (<30, 30–40, 40–50, 50–60, 60–70, ≥70 years) in three population categories: overall (left panel), Chinese (middle panel), and Japanese (right panel), with further stratification by sex. Green bars represent males, red bars represent females, and blue bars represent combined data (All). In all subgroups, incidence rates show a basically consistent increase with advancing age, with peak rates observed in the ≥70 age group (female) and 50-60 age group (male) in overall and Chinese populations, along with peak rates in 60-70 age group in Japanese population. Males demonstrated higher incidence rates than females across all age groups and ethnicities excluding the ≥70 age group in overall and Chinese populations. Japanese populations exhibited substantially higher diabetes incidence in middle-aged and elderly populations between 30-70 years compared to Chinese populations.

**Figure 4** delineates diabetes incidence rates stratified by age, sex, and ethnicity, revealing a consistent age-dependent increase across demographic subgroups. Japanese participants exhibit significantly higher diabetes incidence compared to Chinese participants in both sexes. This ethnic divergence is most pronounced in the middle and older age brackets (30-70 years), with Japanese males aged 60-70 years experiencing peak incidence rates approaching 5.31%. The analysis highlights age progression, male sex, and Japanese ethnicity as critical determinants of diabetes risk within Asian population demographics.

**Table 2** comprehensively details diabetes incidence across AST to ALT ratio quartiles. The study cohort of 99,572 participants yielded 1,403 new diabetes cases during follow-up. This result in an overall incidence rate of 1.4% (95% CI: 1.3-1.5%), corresponding to a cumulative incidence rate of 3.93 per 1,000 person-years. This analysis provides insights into diabetes progression within the studied population.

**Table 2:** Incidence rate of incident diabetes.

AST/ALT	Participants (n)	Diabetes events (n)	Incidence rate (95% CI) (%)	Cumulative incidence (Per 1000 person-year)
All participants				
Total	99572	1403	1.4%(1.3%-1.5%)	3.93
Q1(<0.8666)	24847	648	2.6%(2.4%-2.8%)	6.86
Q2(0.8666-1.1481)	24910	348	1.4%(1.3%-1.5%)	3.76
Q3(1.1481-1.4749)	24920	247	1.0%(0.9%-1.1%)	2.81
Q4(≥1.4749)	24895	160	0.6%(0.5%-0.7%)	1.95
P for trend			<0.001	
Chinese participants				
Total	84281	1032	1.2%(1.2%-1.3%)	3.90
Q1(<0.8886)	21035	427	2.0%(1.8%-2.2%)	6.51
Q2(0.8886-1.1817)	20957	290	1.4%(1.2%-1.5%)	4.39
Q3(1.1817-1.5173)	21218	185	0.9%(0.7%-1.0%)	2.76
Q4(≥1.5173)	21071	130	0.6%(0.5%-0.7%)	1.97
P for trend			<0.001	
Japanese participants				
Total	15291	371	2.4%(2.2%-2.7%)	4.01
Q1(<0.7742)	3691	196	5.3%(4.6%-6.0%)	8.32
Q2(0.7742-0.9830)	3493	74	2.1%(1.6%-2.6%)	3.44
Q3(0.9830-1.2334)	4231	64	1.5%(1.1%-1.9%)	2.54
Q4(≥1.2334)	3876	37	1.0%(0.6%-1.3%)	1.66
P for trend			<0.001	

AST, aspartate aminotransferase; ALT, alanine aminotransferase.

A pronounced dose-response correlation emerged between AST/ALT quartiles and diabetes incidence (p for trend <0.001). Incidence rates showed a consistent downward trend. Specifically, the incidence was 2.6% (95% CI: 2.4-2.8%) in the lowest quartile (Q1:<0.8666), decreased to 1.4% (95% CI: 1.3-1.5%) in Q2, further declined to 1.0% (95% CI: 0.9-1.1%) in Q3, and reached 0.6% (95% CI: 0.5-0.7%) in the highest quartile (Q4: ≥1.4749). Correspondingly, cumulative incidence decreased markedly, from 6.86 to 1.95 per 1000 person-years from Q1 to Q4.

Based on these findings, ethnic stratification revealed distinct diabetes incidence patterns. Chinese participants (n = 84,281) demonstrated an overall incidence of 1.2% (95% CI: 1.2-1.3%), with quartile-based rates decreasing from 2.0% in Q1 to 0.6% in Q4 (p for trend < 0.001). In contrast, Japanese participants (n = 15,291) exhibited a markedly higher overall incidence of 2.4% (95% CI: 2.2-2.7%), characterized by a more pronounced gradient, with rates declining sharply from 5.3% in Q1 to 1.0% in Q4 (p for trend < 0.001).

**Figure 5:**
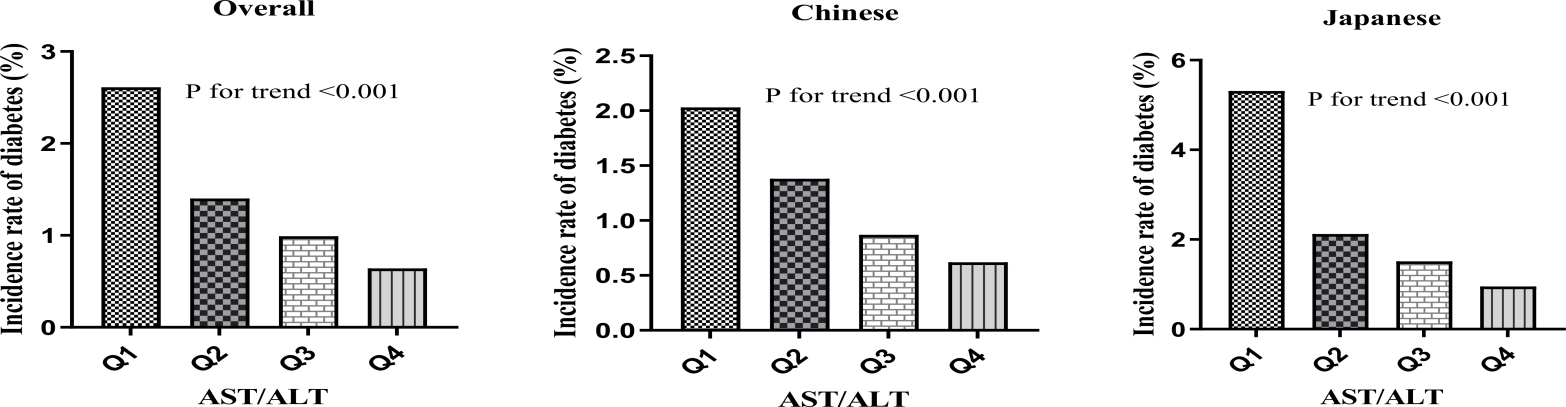
Diabetes incidence by AST/ALT quartiles. Bar charts showing diabetes incidence (%) by AST/ALT quartiles in **(A)** the overall population, **(B)** the Chinese population, and **(C)** the Japanese population. All populations demonstrated a significant negative association between AST/ALT quartiles and diabetes incidence (P for trend<0.001 for all groups), with the Japanese population showing the most pronounced gradient.

Overall, the results illuminate an inverse relationship between AST/ALT values and diabetes risk, revealing a more pronounced gradient in Japanese participants compared to their Chinese counterparts. This pattern is illustrated in **Figure 5**.

### The results of univariate analyses using Cox proportional-hazards regression model

Univariate Cox regression analysis (**Table 3**) unveiled multifaceted diabetes risk factors. Demographic insights showed Japanese participants exhibited lower risk compared to Chinese participants (HR: 0.282), while males displayed heightened risk relative to females (HR: 1.858). Age progression was positively correlated with diabetes development (HR: 1.069 per year). Lifestyle factors, including smoking (HR: 1.556) and alcohol consumption (HR: 1.212), demonstrated significant risk associations. Clinical parameters-–encompassing BMI (HR: 1.244), AST/ALT (HR:0.329), FPG (HR:11.121), specific blood pressure measures, and detailed lipid profiles-–all exhibited statistically significant correlations with diabetes incidence (p < 0.00001).

**Table 3:** The results of univariate analysis.

Index	Statistics	HR	95% CI	P-value
Age	42.036 ± 12.060	1.069	(1.065, 1.074)	<0.00001
Gender				
Female	43264 (43.450%)	Ref.		
Male	56308 (56.550%)	1.858	(1.655, 2.085)	<0.00001
BMI	23.050 ± 3.301	1.244	(1.231, 1.258)	<0.00001
SBP	118.291 ± 16.165	1.039	(1.037, 1.042)	<0.00001
DBP	73.634 ± 10.772	1.050	(1.046, 1.055)	<0.00001
FPG	4.927 ± 0.549	11.121	(9.798, 12.623)	<0.00001
TC	4.751 ± 0.906	1.339	(1.269, 1.412)	<0.00001
TG	1.255 ± 0.953	1.286	(1.267, 1.305)	<0.00001
HDL-c	1.383 ± 0.312	0.355	(0.299, 0.421)	<0.00001
LDL-c	2.767 ± 0.701	1.331	(1.244, 1.424)	<0.00001
Smoking status				
Non-smoker	75721 (76.046%)	Ref.		
Smoker	23851 (23.954%)	1.556	(1.397, 1.734)	<0.00001
Drinking status				
Non-drinker	80863 (81.211%)	Ref.		
Drinker	18709 (18.789%)	1.212	(1.073, 1.370)	0.00201
AST/ALT	1.201 ± 0.443	0.329	(0.283, 0.382)	<0.00001
Country				
Chinese	84281 (84.643%)	Ref.		
Japanese	15291 (15.357%)	0.282	(0.236, 0.337)	<0.00001

BMI, body mass index; SBP, systolic blood pressure; DBP, diastolic blood pressure; FPG, fasting plasma glucose; TC, total cholesterol; TG, triglyceride; HDL-c, high-density lipoprotein cholesterol; LDL-c, low-density lipid cholesterol; AST, aspartate aminotransferase; ALT, alanine aminotransferase.

HR, Hazard ratios; CI, confidence interval, Ref, reference.

**Figure 6:**
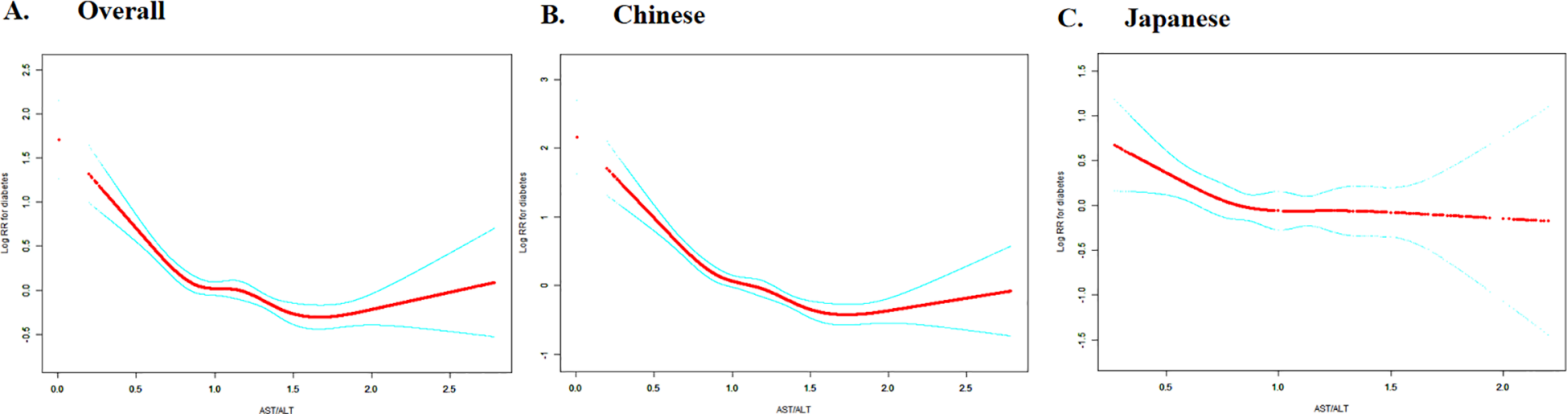
Kaplan-Meier curves for diabetes-free survival stratified by quartiles. The figure analysis stratified by AST/ALT quartiles demonstrated significant associations between AST/ALT and cumulative hazard. In the overall cohort **(A)**, the highest quartile (Q4) showing the lowest cumulative hazard. This pattern was consistently maintained when analyzing ethnic subgroups separately **(B, C)**.

Kaplan-Meier survival analysis (**Figure 6**) revealed strong correlations between AST/ALT and cumulative hazard. Specifically, the overall cohort (Panel A) exhibited progressively lower cumulative hazard across increasing AST/ALT quartiles (p < 0.0001), with the highest quartile (Q4) demonstrating the most favorable outcomes. Furthermore, this systematic trend remained consistent across ethnic subgroups (Panels B and C, p < 0.0001), underscoring the robust inverse relationship between AST/ALT ratio and diabetes risk.

We additionally constructed Kaplan-Meier survival curves for the total population with country as the stratification factor (see **Supplementary Figure 1**), the curves demonstrate that, as follow-up time progresses, the cumulative HRs of developing diabetes in the Chinese cohort is markedly higher than in the Japanese cohort. (p < 0.0001).

### Results from a multivariate Cox proportional-hazards regression model

**Table 4** elucidates the intricate relationship between AST/ALT and diabetes risk through progressively refined statistical models across ethnic populations.

**Table 4:** Relationship between AST/ALT and the incident diabetes in different models.

AST/ALT exposure	Overall (HR,95%CI, P)	Chinese (HR,95%CI, P)	Japanese (HR,95%CI, P)
Model I:			
AST/ALT	0.329 (0.283, 0.382) <0.00001	0.323 (0.275, 0.379) <0.00001	0.129 (0.088, 0.189) <0.00001
AST/ALT quartiles			
Q1	Ref.	Ref.	Ref.
Q2	0.557 (0.489, 0.635) <0.00001	0.656 (0.565, 0.762) <0.00001	0.428 (0.327, 0.559) <0.00001
Q3	0.445 (0.384, 0.515) <0.00001	0.415 (0.349, 0.493) <0.00001	0.325 (0.245, 0.431) <0.00001
Q4	0.342 (0.287, 0.407) <0.00001	0.302 (0.248, 0.368) <0.00001	0.221 (0.155, 0.315) <0.00001
P for trend	<0.00001	<0.00001	<0.00001
Model II:			
AST/ALT	0.433 (0.362, 0.517) <0.00001	0.365 (0.298, 0.446) <0.00001	0.338 (0.219, 0.521) <0.00001
AST/ALT quartiles			
Q1	Ref.	Ref.	Ref.
Q2	0.638 (0.556, 0.731) <0.00001	0.625 (0.534, 0.731) <0.00001	0.570 (0.432, 0.750) 0.00006
Q3	0.551 (0.469, 0.648) <0.00001	0.451 (0.373, 0.546) <0.00001	0.545 (0.402, 0.739) 0.00009
Q4	0.474 (0.389, 0.578) <0.00001	0.381 (0.304, 0.477) <0.00001	0.482 (0.328, 0.709) 0.00021
P for trend	<0.00001	<0.00001	<0.00001
Model III:			
AST/ALT	0.539 (0.452, 0.643) <0.00001	0.417 (0.341, 0.510) <0.00001	0.631 (0.416, 0.956) 0.02972
AST/ALT quartiles			
Q1	Ref.	Ref.	Ref.
Q2	0.696 (0.607, 0.799) <0.00001	0.632 (0.539, 0.740) <0.00001	0.735 (0.557, 0.970) 0.02942
Q3	0.627 (0.533, 0.738) <0.00001	0.478 (0.395, 0.578) <0.00001	0.814 (0.599, 1.108) 0.19105
Q4	0.599 (0.491, 0.730) <0.00001	0.435 (0.348, 0.545) <0.00001	0.717 (0.488, 1.054) 0.09075
P for trend	<0.00001	<0.00001	0.05561

Model I: we did not adjust other covariates.

Model II: we adjust age, gender, BMI, SBP, DBP, smoking and drinking status.

Model III: we adjust age, gender, BMI, SBP, DBP, FPG, LDL-c, HDL-c, TG, smoking and drinking status.

HR, Hazard ratios; CI: confidence, Ref: reference; AST, aspartate aminotransferase; ALT, alanine aminotransferase.

In the unadjusted model (Model I), AST/ALT demonstrated a robust inverse association with diabetes risk. The analysis of continuous variable revealed stronger inverse association in Japanese participants (HR: 0.129, 95% CI: 0.088-0.189) compared to Chinese participants (HR: 0.323, 95% CI: 0.275-0.379). Analyses based on quartiles consistently showed substantial risk reduction in the highest quartile (Q4) across all populations, indicating statistically significant dose-response relationships.

In Model II, adjusted for demographic and lifestyle factors, the associations remained significant. The continuous variable analysis, and quartile comparisons, sustained the inverse risk relationship, with slightly attenuated but still meaningful HRs. The continuous variable analysis revealed HRs of 0.433 (95% CI: 0.362-0.517), 0.365 (95% CI: 0.298-0.446), and 0.338 (95% CI: 0.219-0.521) in the overall, Chinese, and Japanese populations, respectively. Quartile-stratified analyses consistently showed that the Q4 versus Q1 comparison yielded HRs of 0.474 (95% CI: 0.389-0.578), 0.381 (95% CI: 0.304-0.477), and 0.482 (95% CI: 0.328-0.709) for the respective populations.

The fully adjusted model (Model III), which incorporated metabolic parameters, revealed persistent yet moderately attenuated associations. The continuous variable analysis revealed HRs of 0.539 (95% CI: 0.452-0.643), 0.417 (95% CI: 0.341-0.510), and 0.631 (95% CI: 0.416-0.956) in the overall, Chinese, and Japanese populations, respectively. Quartile-stratified analyses in the overall and Chinese populations were consistent (P for trend < 0.05). Japanese participants exhibited less definitive associations, approaching but not reaching the conventional significance threshold of 0.05 (P for trend = 0.05561).

### Sensitivity analysis

**Table 5** explores the robustness of the relationship between AST/ALT and diabetes incidence through meticulously designed sensitivity analyses. The study constructs targeted models to systematically investigate potential confounding variables across diverse population subgroups.

**Table 5:** Relationship between AST/ALT and diabetes in different sensitivity analyses.

Exposure	Model I (HR,95%CI, P)	Model II (HR,95%CI, P)	Model III (HR,95%CI, P)	Model IV (HR,95%CI, P)
AST/ALT	0.563 (0.462, 0.686) <0.00001	0.544 (0.439, 0.673) <0.00001	0.522 (0.428, 0.637) <0.00001	0.604 (0.483, 0.754) <0.00001
AST/ALT (Quartile)				
Q1	Ref.	Ref.	Ref.	Ref.
Q2	0.668 (0.568, 0.785) <0.00001	0.697 (0.582, 0.834) 0.00008	0.677 (0.577, 0.795) <0.00001	0.731 (0.608, 0.879) 0.00088
Q3	0.630 (0.524, 0.757) <0.00001	0.563 (0.459, 0.690) <0.00001	0.568 (0.471, 0.685) <0.00001	0.610 (0.493, 0.755) <0.00001
Q4	0.565 (0.453, 0.704) <0.00001	0.553 (0.437, 0.701) <0.00001	0.572 (0.459, 0.713) <0.00001	0.608 (0.475, 0.778) 0.00008
P for trend	<0.00001	<0.00001	<0.00001	<0.00001

Model I was sensitivity analysis in participants without BMI≥28kg/m^2^ (N = 91,934). We adjusted age, gender, BMI, SBP, DBP, FPG, LDL-c, HDL-c, TG, smoking and drinking status.

Model II was a sensitivity analysis performed on never smoker participants (N = 75,721). We adjusted age, gender, BMI, SBP, DBP, FPG, LDL-c, HDL-c, TG, drinking status.

Model III was a sensitivity analysis performed on never drinker participants (N = 80,863). We adjusted age, gender, BMI, SBP, DBP, FPG, LDL-c, HDL-c, TG, smoking status.

Model IV was sensitivity analysis in participants without TG≥1.7mmol/L (N = 79,202). We adjusted age, gender, BMI, SBP, DBP, FPG, LDL-c, HDL-c, TG, smoking and drinking status.

HR, Hazard ratios; CI: confidence, Ref: reference; AST, aspartate aminotransferase; ALT, alanine aminotransferase.

Sensitivity analyses revealed the remarkable consistency of the inverse association between AST/ALT and diabetes risk across diverse population subgroups. Notably, the inverse relationship remained statistically significant in non-obese individuals (HR = 0.563, 95% CI: 0.462-0.686), never-smokers (HR = 0.544, 95% CI: 0.439-0.673), never-drinkers (HR = 0.522, 95% CI: 0.428-0.637), and participants with normal TG levels (HR = 0.604, 95% CI: 0.483-0.754). Each model demonstrated a robust, statistically significant inverse association between AST/ALT and diabetes incidence (P < 0.00001), underscoring the relationship’s reliability across varied demographic and metabolic contexts.

Quartile-based analysis consistently revealed a robust dose-response relationship across all sensitivity models (P for trend < 0.00001). The highest quartile (Q4) systematically demonstrated a significantly reduced diabetes risk compared to the lowest quartile (Q1). This protective effect was evident across diverse subgroups: non-obese participants (HR = 0.565, 95% CI: 0.453-0.704), never smokers (HR = 0.553, 95% CI: 0.437-0.701), never drinkers (HR = 0.572, 95% CI: 0.459-0.713), and individuals with normal TG levels (HR = 0.608, 95% CI: 0.475-0.778), all with statistically significant risk reduction (P < 0.00001).

Additionally, considering subclinical diabetes and other metabolic diseases, as well as the substantial missing data on smoking and drinking status, we further conducted sensitivity analyses by excluding incident cases in the first 2 years and without adjusting for smoking and drinking status, showing the inverse association between AST/ALT and diabetes risk consistently (see **Supplementary Table S3**).

### The non-linearity addressed by Cox proportional hazards regression model with cubic spline functions

**Figure 7:**
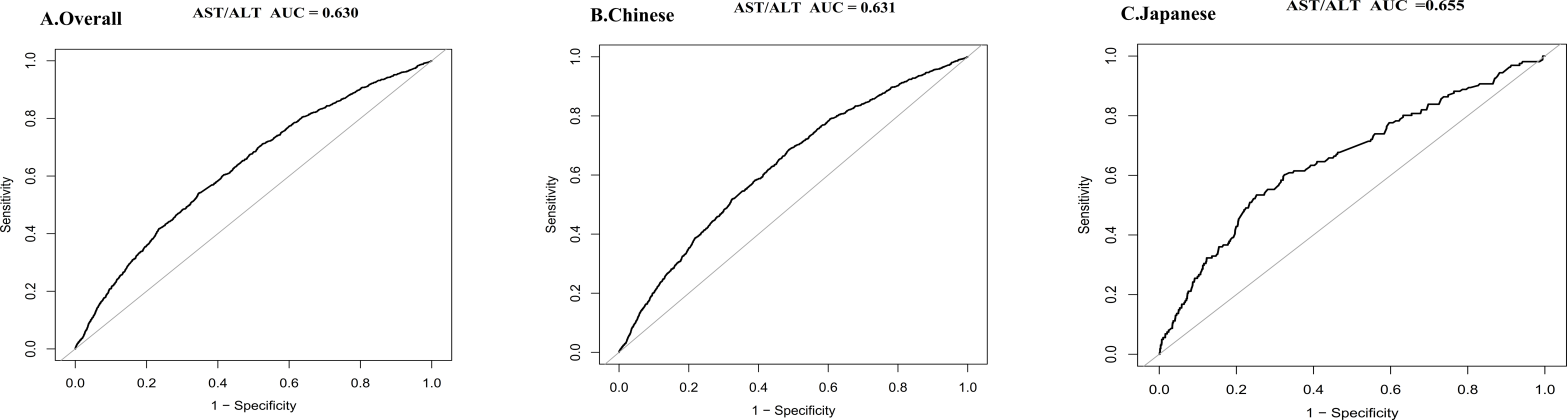
Dose-response relationship between AST/ALT and diabetes risk with ethnic-specific threshold effects. The figure shows restricted cubic spline curves depicting the dose-response relationship between AST/ALT and logarithm of relative risk (Log RR) for diabetes. Panel A presents data for the overall study population, while Panel B and C shows ethnicity-stratified analyses for Chinese (left) and Japanese (right) cohorts. The solid lines represent the estimated Log RR values. Analyses based on overall and Chinese populations demonstrate negative associations between AST/ALT and diabetes risk, with evidence of non-linearity.

**Figure 7** reveals the nuanced, non-linear dynamics of the relationship between AST/ALT ratio and diabetes risk in Asian populations. The restricted cubic spline curves elegantly visualize the intricate dose-response pattern, while the two-piecewise (segmented) Cox regression analysis (**Table 6**) precisely delineates the complex association, identifying distinct inflection points that differentiate ethnic-specific risk trajectories.

**Table 6:** The result of the two-piecewise Cox regression model.

Incident diabetes	Overall (HR,95%CI, P)	Chinese (HR,95%CI, P)	Japanese (HR,95%CI, P)
Fitting model by standard Cox regression	0.539 (0.452, 0.643) <0.0001	0.417 (0.341, 0.510) <0.0001	0.631 (0.416, 0.956) 0.0297
Fitting model by two-piecewise Cox regression			
Inflection point of AST/ALT	0.882	0.912	0.882
≤Inflection point	0.174 (0.110, 0.276) <0.0001	0.110 (0.065, 0.186) <0.0001	0.320 (0.140, 0.730) 0.0068
>Inflection point	0.768 (0.620, 0.951) 0.0154	0.627 (0.492, 0.798) 0.0002	0.999 (0.540, 1.847) 0.9969
P for log-likelihood ratio test	<0.001	<0.001	0.065

We adjusted age, gender, BMI, SBP, DBP, FPG, LDL-c, HDL-c, TG, smoking and drinking status.

HR, Hazard ratios; CI: confidence, Ref: reference; AST, aspartate aminotransferase; ALT, alanine aminotransferase.

The overall population analysis revealed a statistically significant non-linear relationship between the AST/ALT ratio and diabetes risk, with a critical inflection point at 0.882 (p for log-likelihood ratio test <0.001). Below this threshold, each one unit increase in the AST/ALT ratio was associated with an 82.6% reduction in diabetes risk (HR = 0.174, 95% CI: 0.110-0.276). Conversely, beyond this point, the effect diminished, showing a more modest but still significant risk reduction (HR = 0.768, 95% CI: 0.620-0.951).

Extending this analysis to specific ethnic groups revealed distinct non-linear patterns in the relationship between the AST/ALT ratio and diabetes risk. The Chinese cohort mirrored the overall population trend, with an inflection point at 0.912. Below this threshold, a robust effect was observed (HR = 0.110, 95% CI: 0.065-0.186), which substantially weakened above the point (HR = 0.627, 95% CI: 0.492-0.798). In contrast, the Japanese cohort displayed a more pronounced divergence, with an inflection point at 0.882. The association was significant below the threshold (HR = 0.320, 95% CI: 0.140-0.730) but became statistically indistinguishable above it (HR = 0.999, 95% CI: 0.540-1.847). These findings highlight the nuanced ethnic variations in this metabolic relationship.

### The results of subgroup analyses

**Table 7** elucidates the intricate relationship between AST/ALT and diabetes incidence across diverse population subgroups. Following covariate adjustment, the analysis revealed pronounced effect modification by key demographic and physiological parameters, with age, ethnicity, and SBP playing influential roles in modulating the AST/ALT-diabetes risk association.

**Table 7:** Effect size of AST/ALT on incident diabetes in prespecified and exploratory subgroups.

Characteristic	No of participants	HR (95%CI)	P value	P for interaction
Age(years)				<0.0001
20 to <45	64200	0.303 (0.208, 0.442)	<0.0001	
45 to <60	25200	0.688 (0.526, 0.898)	0.006	
≥60	10167	0.841 (0.635, 1.113)	0.2265	
Gender				0.8256
Male	56308	0.540 (0.433, 0.673)	<0.0001	
Female	43264	0.561 (0.425, 0.740)	<0.0001	
BMI (kg/m^2^)				0.896
<18.5	6366	0.887 (0.232, 3.397)	0.8608	
≥18.5, <24	56673	0.546 (0.408, 0.730)	<0.0001	
≥24, <28	28895	0.518 (0.396, 0.678)	<0.0001	
≥28	7638	0.532 (0.354, 0.800)	0.0024	
Smoking status				0.8019
Non-smoker	75721	0.549 (0.446, 0.675)	<0.0001	
Smoker	23851	0.524 (0.387, 0.710)	<0.0001	
Drinking status				0.3924
Non-drinker	80863	0.520 (0.428, 0.632)	<0.0001	
Drinker	18709	0.619 (0.432, 0.886)	0.0087	
SBP (mmHg)				0.0431
<140	90389	0.486 (0.396, 0.596)	<0.0001	
≥140	9183	0.718 (0.520, 0.990)	0.0434	
DBP (mmHg)				0.9829
<90	92251	0.539 (0.446, 0.652)	<0.0001	
≥90	7321	0.542 (0.355, 0.827)	0.0045	
Country				0.0434
Chinese	84281	0.404 (0.332, 0.492)	<0.0001	
Japanese	15291	0.636 (0.427, 0.947)	0.026	

Note 1: Above model adjusted for age, gender, BMI, SBP, DBP, FPG, LDL-c, HDL-c, TG, smoking and drinking status.

Note 2: In each case, the model is not adjusted for the stratification variable.

Age-stratified analysis revealed a progressive attenuation of the AST/ALT-diabetes risk relationship with increasing age (P for interaction<0.0001). The AST/ALT-diabetes risk relationship was most robust in younger adults (20-45 years, HR: 0.303, 95% CI: 0.208-0.442) and moderately strong in middle-aged participants (45-60 years, HR: 0.688, 95% CI: 0.526-0.898). However, this association became markedly diminished and statistically insignificant among individuals aged 60 and above (HR: 0.841, 95% CI: 0.635-1.113).

Ethnic variation significantly influenced the AST/ALT-diabetes risk association (P for interaction = 0.0434). The AST/ALT-diabetes risk relationship was markedly more pronounced in Chinese populations (HR = 0.404, 95% CI: 0.332-0.492, P < 0.0001), while Japanese populations exhibited a comparatively attenuated association (HR: 0.636, 95% CI: 0.427-0.947, P = 0.026), highlighting the complex ethnic-specific metabolic interactions underlying diabetes risk.

SBP emerged as a significant modifier of the AST/ALT-diabetes risk relationship (P for interaction = 0.0431). The AST/ALT-diabetes risk relationship was substantially more pronounced in normotensive individuals (SBP < 140 mmHg, HR: 0.486, 95% CI: 0.396-0.596), while showing a notably reduced impact among hypertensive participants (SBP ≥ 140 mmHg, HR = 0.718, 95% CI: 0.520-0.990), suggesting blood pressure’s crucial role in mediating this metabolic association.

No significant effect modification was observed for gender, BMI, smoking status, drinking status, or DBP (all P for interaction > 0.05).

### Predictive performance of AST/ALT across populations

**Figure 8:**
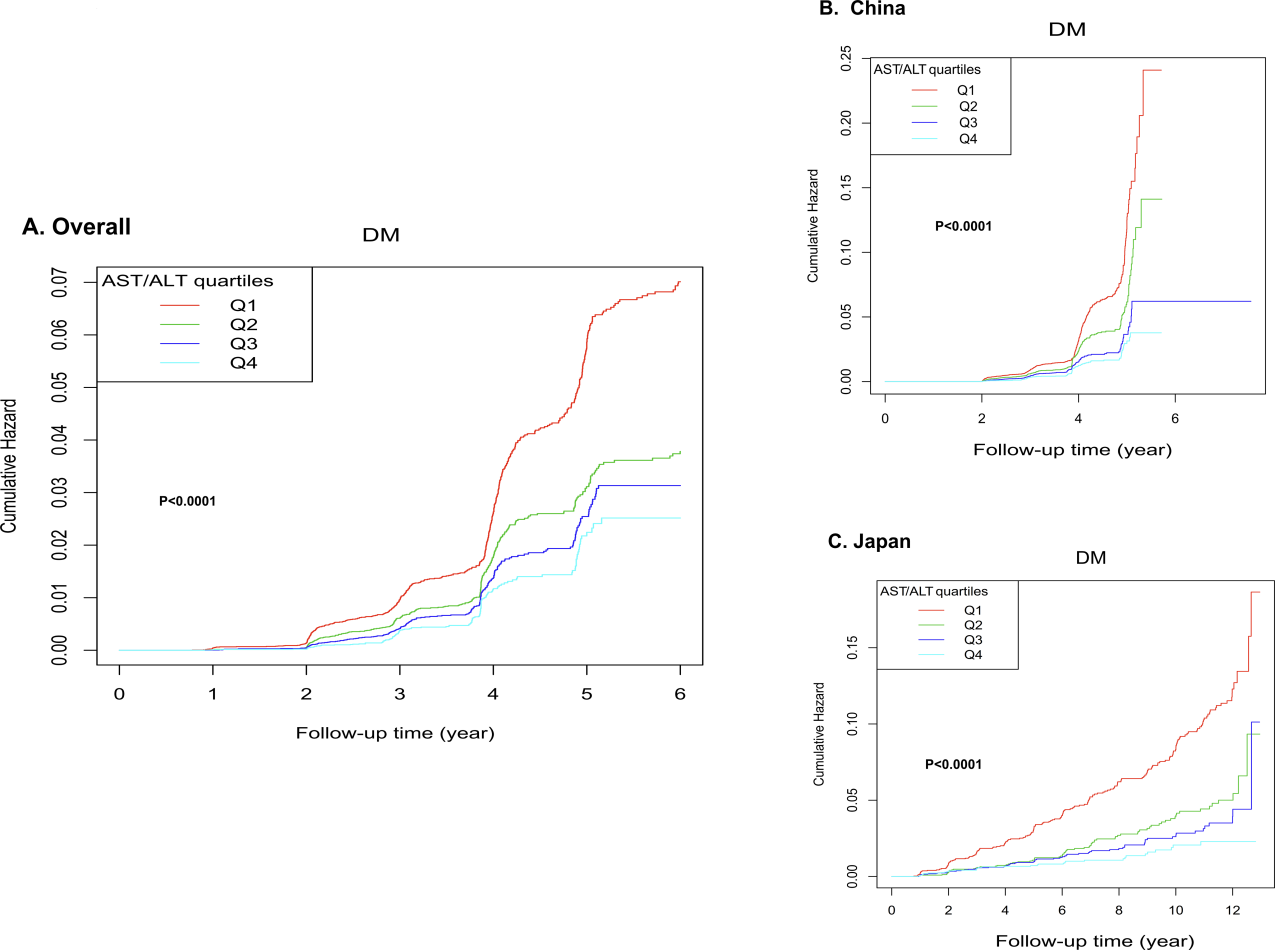
Discriminative performance of the AST/ALT for diabetes risk in overall, Chinese, and Japanese populations. ROC curve illustrating the predictive value of AST/ALT in the overall study cohort (AUC = 0.630) **(A)**, in the Chinese subgroup (AUC = 0.631) **(B)**, and in the Japanese subgroup (AUC = 0.655) **(C)**. Sensitivity and 1-specificity values are plotted to assess the diagnostic performance of AST/ALT in identifying individuals at increased risk of diabetes. AUC values were calculated to quantify the overall accuracy of AST/ALT in each population. All analyses were conducted using standard ROC analysis methods. Higher AUC values are indicative of better discriminative capability.

The predictive performance of AST/ALT varied across populations, revealing a moderate discriminative capacity in the overall cohort (AUC = 0.630). Chinese participants showed a comparable predictive ability (AUC = 0.631), while the Japanese subgroup demonstrated enhanced discriminative power (AUC = 0.655). These results suggest AST/ALT’s potential as a refined diabetes risk marker, with particular promise in the Japanese population context (see **Figure 8**).

### BMI partially mediates the association between AST/ALT and incident diabetes

**Table 8** presents the mediation analysis that explores BMI’s role in the AST/ALT-diabetes risk pathway. Following comprehensive covariate adjustment, the analysis reveals substantial direct and indirect effects, demonstrating BMI’s complex mediating role across diverse population cohorts in modulating the relationship between AST/ALT and diabetes incidence.

**Table 8:** Mediation analysis of the effect of BMI on the AST/ALT-diabetes relationship.

Effect	Total population	Chinese population	Japanese population
	HR (95% CI)	HR (95% CI)	HR (95% CI)
Direct effect (AST/ALT→Diabetes)	0.54(0.45-0.64)	0.42(0.34-0.51)	0.63(0.42-0.96)
Indirect effect (AST/ALT→BMI→Diabetes)	0.75(0.73-0.78)	0.77(0.74-0.80)	0.78(0.73-0.83)
Total effect (AST/ALT→Diabetes)	0.38(0.32-0.45)	0.29(0.24-0.35)	0.46(0.30-0.70)
Proportion mediated	29.09% (23.60% - 37.17%)	20.96%(16.33%-27.41%)	32.22%(17.77%-79.58%)

Across the total population, AST/ALT demonstrated a notable direct impact on diabetes risk (HR: 0.54, 95% CI: 0.45-0.64), complemented by a significant indirect effect mediated through BMI (HR: 0.75, 95% CI: 0.73-0.78). The cumulative total effect resulted in a HR of 0.38 (95% CI: 0.32-0.45), with BMI accounting for 29.09% of the total effect, highlighting the complex interplay between AST/ALT, BMI, and diabetes risk.

Ethnic stratification revealed nuanced mediation dynamics. In the Chinese cohort, AST/ALT’s direct effect on diabetes risk (HR: 0.42, 95% CI: 0.34-0.51) was accompanied by a significant BMI-mediated indirect effect (HR: 0.77, 95% CI: 0.74-0.80), with BMI mediating 20.96% of the total effect (HR: 0.29, 95% CI: 0.24-0.35). The Japanese subgroup exhibited a distinct pattern, featuring a direct effect (HR: 0.63, 95% CI: 0.42-0.96) and a more substantial BMI-mediated effect of 32.22% (indirect effect HR: 0.78, 95% CI: 0.73-0.83), underscoring the population-specific metabolic interactions underlying diabetes risk.

## Discussion

Our investigation uncovered a significant inverse relationship between AST/ALT ratio and diabetes incidence across Chinese and Japanese populations. It is important to distinguish our findings (about the ratio of AST/ALT) from studies examining elevated levels of individual enzymes, which are typical markers of liver cell injury. After rigorous adjustment for confounder, this association remained consistent. Moreover, a dose-response gradient was observed, with higher AST/ALT quartiles correlating with reduced diabetes risk. Additionally, non-linear patterns with population-specific threshold effects and modifying influences from age, ethnicity, and SBP emerged. Critically, mediation analysis revealed that BMI partially mediated this relationship, thereby weakening the association between high AST/ALT and diabetes prevention.

Our research revealed distinct diabetes incidence patterns between the Chinese and Japanese populations. The Chinese cohort exhibited a lower incidence rate of 1.2%, contrasting sharply with the Japanese population’s 2.4%. This disparity was consistently observed across age groups, with the most pronounced differences emerging in middle-aged and elderly individuals (30-70 years). Notably, Japanese males aged 60-70 years demonstrated the peak incidence rate at approximately 5.31%. These divergent rates likely result from complex interactions among dietary habits, lifestyle factors, and genetic predispositions unique to each population (57). The Japanese dietary pattern, while rich in fish and vegetables, also includes substantial amounts of refined carbohydrates such as white rice, which may contribute to significant postprandial glucose fluctuations and specific metabolic disturbances, including impaired insulin sensitivity and lipid metabolism (58). Furthermore, Japanese societal characteristics-–marked by intense work environments and sedentary lifestyles-–independently contribute to elevated diabetes risk, potentially exacerbating metabolic vulnerability through chronic stress and reduced physical activity (59). Genetic studies have identified specific polymorphisms in the TCF7L2 and KCNQ1 gene loci among Japanese populations, which may intrinsically increase diabetes susceptibility via distinct molecular mechanisms (60). Additionally, differences in follow-up duration may partially explain the disparate diabetes incidence rates; the Chinese cohort was followed for approximately 7 years, whereas the Japanese population underwent a longer 12-year surveillance period, potentially leading to higher observed incidence due to extended observation time.

The dose-response relationship between AST/ALT quartiles and diabetes incidence exhibited a more pronounced gradient in the Japanese cohort, with a dramatic decline in diabetes incidence from 5.3% in Q1 to 1.0% in Q4. In contrast, the Chinese population showed a more modest reduction from 2.0% to 0.6%. This pattern suggests a potentially greater metabolic sensitivity to AST/ALT variations among Japanese individuals, likely due to distinctive lipid metabolism and physiological characteristics (61).

After comprehensive adjustment for confounders, our analysis revealed a robust inverse correlation between AST/ALT and diabetes risk. As one of the well-known marker of hepatic steatosis (22, 23, 62), low AST/ALT typically signify hepatic fat deposition, which correlates closely with worsening insulin resistance, impaired pancreatic β-cell function, and escalating glycemic dysregulation (35, 63). In the fully adjusted model, our findings supported existing evidence, showing that each unit increase in AST/ALT corresponded to a substantial reduction in diabetes risk: 46.1% in the overall population, a more pronounced 58.3% in the Chinese cohort, and a notable 36.9% decrease in the Japanese population.

Interestingly, subgroup interaction testing and univariate Cox analysis revealed significant differences between countries (P = 0.0434), with the Japanese cohort showing substantially lower risk (HR: 0.282 for Japanese vs Chinese). Correspondingly, Kaplan-Meier survival curves demonstrated that the cumulative HR of developing diabetes was markedly higher in the Chinese cohort throughout the follow-up period.

These indicate population-specific variations in the AST/ALT-diabetes risk relationship that merit further investigation. Despite the insights gained from subgroup and interaction analyses, the disparity in sample sizes between the Chinese and Japanese cohorts may restrict the robustness of subgroup analyses, particularly for the smaller Japanese group. This limitation affects the precision of subgroup estimates (reflected in wider confidence intervals) and reduces the statistical power for interaction tests, which typically require larger sample sizes than main effect analyses. These observed differences may be attributed to multiple factors, including genetic, environmental, and lifestyle influences. Firstly, genetic predispositions might play a significant role in how different populations metabolize liver enzymes and their relationship with diabetes. Variations in genes related to liver function, insulin sensitivity, and glucose metabolism could lead to differences in AST to ALT ratio as well as their separate implications for diabetes risk (64, 65). Secondly, the dietary habits and lifestyle choices of the Japanese and Chinese populations differ significantly. For instance, the Japanese diet is often lower in carbohydrates and higher in fish and vegetables, which may influence liver enzyme levels and their association with diabetes. In contrast, the Chinese diet may include higher carbohydrate intake and different types of fats, potentially affecting metabolic health and liver function (66). Furthermore, differences in healthcare access and screening practices might also contribute to the observed disparities. The Japanese healthcare system emphasizes the importance of regular health check-ups, which may lead to earlier detection and management of diabetes and related conditions. In contrast, variations in healthcare access in China could result in differences in the identification and treatment of diabetes, potentially affecting the association with liver enzymes (67, 68).

Sensitivity analyses reinforced the consistent negative correlation between AST/ALT and diabetes risk. This association remained statistically significant even after excluding potential confounding subgroups such as obese individuals, smokers, drinkers, and those with hypertriglyceridemia. These findings highlight the significant predictive value of AST to ALT ratio for diabetes risk. Subgroup analyses identified additional important effect modifiers beyond differences between countries. Age emerged as a critical determinant, demonstrating a statistically significant interaction (P < 0.0001), the robust AST/ALT-diabetes risk correlations were prominently evident in young (20 - 45 years) and middle-aged (45 - 60 years) cohorts, while losing significance among elderly populations (≥ 60 years). Similarly, SBP exhibited a notable interaction (P = 0.0431), revealing more pronounced associations in individuals maintaining pressures below 140 mmHg.

Emerging research across diverse ethnic populations has consistently illuminated a nuanced, non-linear relationship between AST/ALT ratio and diabetes incidence (28, 31, 32, 69). However, diabetes risk assessment models derived from specific ethnic contexts may lack universal translatability across different population groups (70). Employing restricted cubic spline curves and piecewise Cox regression models, we unveiled a complex non-linear relationship between AST/ALT and diabetes risk. We further identified population-specific inflection dynamics. In the aggregate cohort, the critical point at AST/ALT= 0.882 demarcated distinct risk patterns: below this threshold, each AST/ALT unit increment correlated with an impressive 82.6% diabetes risk reduction (HR = 0.174, P < 0.0001), beyond this point, the risk reduction weakened to approximately 23.2% (HR = 0.768, P = 0.0154). The Chinese population mirrored this pattern, with an inflection point at AST/ALT= 0.912, showing a more pronounced risk reduction below this threshold (HR = 0.110, P < 0.0001) and a diminished risk reduction above it (HR = 0.627, P = 0.0002). Notably, this inflection point diverged from previous research by Xie et al., which suggested a different risk trajectory around an AST/ALT ratio of 1.18 (33). Our analysis of the Japanese population revealed a non-linear relationship with a distinct saturation effect at the AST/ALT inflection point of 0.882. Below this threshold, a significant negative association emerged (HR = 0.320, P = 0.0068), beyond this point, the correlation became statistically insignificant (HR = 0.999, P = 0.9969). This pattern closely aligns with prior research findings, which demonstrated a marked negative correlation for AST/ALT ratios below 0.882 (HR: 0.287, 95% CI: 0.126-0.655, P = 0.003), followed by a pronounced saturation effect where the relationship became statistically indistinguishable (HR: 1.036, 95% CI: 0.561-1.913, P = 0.9105) (32).

The saturation effect observed in both populations indicates that beyond a certain AST/ALT threshold, the strength of AST/ALT-diabetes associations tended to weaken or even disappear. This suggests that other metabolic pathways or compensatory mechanisms may be involved, limiting the impact of liver enzyme levels on diabetes risk at higher AST/ALT ratios. For instance, individuals with higher AST/ALT ratios may already have other metabolic disturbances that overshadow the effect of liver enzymes on diabetes risk. Understanding the non-linear relationship allows for more precise risk stratification in clinical practice. Furthermore, identifying distinct thresholds for AST/ALT ratios in different populations can inform personalized interventions.

This study additionally evaluated AST/ALT’s ability to predict diabetes risk, showing that AST/ALT has good predictive value in both populations. When comparing ROC curves between Chinese and Japanese populations, we found largely consistent predictive performance. However, AST/ALT’s ability to predict diabetes in the Japanese population appeared to be slightly better than in the Chinese population, indicating the AST/ALT’s value as a predictive marker in different Eastern Asian populations.

Mediation analysis revealed BMI’s significant partial mediating role in the relationship between AST/ALT and diabetes development. Across the overall population, BMI mediated 29.09% of the total effect, indicating two mechanisms: an indirect pathway through BMI (AST/ALT affecting BMI, which in turn affects diabetes risk) and a direct pathway independent of BMI (AST/ALT directly affecting diabetes risk). Notably, mediation effects varied between populations: Chinese subjects demonstrated a 20.96% BMI-mediated effect, while Japanese subjects exhibited a more pronounced 32.22% mediation. This suggests that in the Japanese population, the effect of AST/ALT on diabetes risk is more substantially mediated through obesity, whereas in the Chinese population, AST/ALT exerts a more direct influence.

From a physiological standpoint, the precise mechanism linking AST/ALT to diabetes remains unclear. Current hypotheses suggest that low AST/ALT ratios serve as clinical biochemical indicators of hepatic dysfunction, reflecting cellular damage through enzyme release. Emerging evidence indicates that hepatic inflammation disrupts insulin signaling pathways, compromising the liver’s ability to regulate glucose production and ultimately leading to hyperglycemia (24, 71). Systemic insulin resistance triggers a harmful metabolic cascade. Impaired cellular glucose uptake and altered fatty acid metabolism leads to increased gluconeogenesis and lipid synthesis, which compromise liver function. Simultaneously, escalating inflammation and oxidative stress release pro-inflammatory cytokines, further amplifying insulin resistance and creating a self-perpetuating cycle of metabolic dysfunction (72, 73). Fat accumulation and inflammatory processes progressively degrade liver architecture, causing structural disruption and cellular damage, which consequently elevates ALT enzyme levels as a marker of hepatic injury (69). AST, a key enzyme in aerobic metabolism, is distributed across multiple tissues including liver, heart, muscles, kidneys, and red blood cells. During insulin resistance, impaired glucose uptake by cells disrupts aerobic metabolic processes, consequently diminishing AST enzyme activity (74, 75). Finally, The AST/ALT ratio decreases. BMI, despite limitations in precisely measuring fat distribution, offers insights into visceral fat distribution (76). Our observations underscore the significance of AST/ALT as diabetes risk indicators and emphasize BMI control through lifestyle intervention as a potential preventive strategy.

The study reveals that AST/ALT serves as a robust biomarker for diabetes risk prediction in East Asian populations, providing nuanced clinical insights. Furthermore, unique population-specific thresholds (0.912 for Chinese and 0.882 for Japanese) highlight the critical need for tailored screening approaches. Subgroup analyses demonstrate varied effectiveness of specific interventions across demographic segments, particularly among young and middle-aged adults with normal blood pressure. The research underscores the importance of multi-pronged strategies, emphasizing personalized interventions that address metabolic complexities and potential lipid metabolism disorders through comprehensive approaches such as dietary optimization, physical activity, and targeted pharmacological treatments (77).

The study’s strengths lie in its robust methodology: a large prospective cohort design across two East Asian populations with distinct lifestyles but similar genetic backgrounds, which enhances the generalizability of the results. Advanced analytical techniques, including multivariate Cox regression, sensitivity analyses, and mediation analysis, provided comprehensive insights into the relationship between AST/ALT and diabetes risk. Moreover, piecewise regression models further refined the analysis by identifying population-specific thresholds, thereby offering valuable clinical guidance and a nuanced understanding of metabolic risk factors.

The study acknowledges several methodological constraints: firstly, potential residual confounding despite extensive adjustments; secondly, reliance on single baseline AST/ALT measurements without tracking longitudinal changes; and thirdly, disparate sample sizes between Chinese (n = 84,281) and Japanese (n = 15,291) populations. Diagnostic limitations include using FPG alone and self-reported diabetes diagnosis, which may introduce classification bias. Additionally, critical covariate data-–such as family history, diet, and physical activity-–were incompletely captured. Finally, the findings’ applicability is restricted to East Asian populations, limiting broader racial and ethnic generalizability. Therefore, further cross-population research is necessary to validate the observed associations.

The study reveals a significant negative correlation between AST/ALT and diabetes risk in Chinese and Japanese populations, demonstrating robust associations after comprehensive confounder adjustments. Population-specific non-linear relationships emerged, with distinct threshold effects: Japanese populations show diminished risk reduction beyond AST/ALT > 0.882, while Chinese populations maintain significant associations up to AST/ALT > 0.912. These country-specific variations underscore the necessity of targeted risk assessment and intervention strategies. Mediation analysis further illuminated BMI’s partial mediating role in the AST/ALT-diabetes relationship, with notably higher mediation observed in the Japanese population, highlighting the complex interplay of metabolic factors across different ethnic contexts.

## Data Availability

The original contributions presented in the study are included in the article/**Supplementary Material**. Further inquiries can be directed to the corresponding author/s.
