# Supplementary material for: Association between the ratio of aspartate aminotransferase to alanine aminotransferase and risk of diabetes and the mediating effect of BMI: a comparative analysis in Chinese and Japanese populations
Source: Front Endocrinol (Lausanne). 2026 Jan 20;16:1704211. doi: 10.3389/fendo.2025.1704211 (PMC12864127; doi:10.3389/fendo.2025.1704211)
Supplement: Supplementary file 2 [file Table1.docx]

Supplementary Material

**Table S1. Collinearity diagnostics steps.**

|  | Step 1 | Step 2 |
| --- | --- | --- |
| AST/ALT | 1.4 | 1.4 |
| Age | 1.3 | 1.2 |
| Gender | 1.7 | 1.7 |
| BMI | 1.5 | 1.5 |
| SBP | 2.3 | 2.3 |
| DBP | 2.2 | 2.2 |
| FPG | 1.1 | 1.1 |
| TC | 8.7 | NA |
| TG | 1.7 | 1.3 |
| HDL-c | 1.8 | 1.2 |
| LDL-c | 7.2 | 1.1 |
| Smoking status | 1.3 | 1.3 |
| Drinking status | 1.2 | 1.2 |

AST, Aspartate aminotransferase; ALT, Alanine aminotransferase; BMI, Body mass index; SBP, Systolic blood pressure; DBP, Diastolic blood pressure; FPG, Fasting plasma glucose; TC, Total cholesterol; TG, triglyceride; HDL-c, high-density lipoprotein cholesterol; LDL-c, Low-density lipid cholesterol.

Abbreviation: VIF: variance inflation factor; VIF = 1/(1-R^2^).

Note: The variables with VIF>5 will be regarded as collinear variables and cannot be included in the multiple regression model.

**Table S2. Demographic and clinical characteristics of study population by the country**

| **Characteristics** | **Chinese (n=84,281)** | **Japanese(n=15,291)** | **P-value** |
| --- | --- | --- | --- |
| **Age (years)** | 41.73 ± 12.53 | 43.72 ± 8.89 | <0.001 |
| **BMI (kg/m²)** | 23.22 ± 3.30 | 22.13 ± 3.13 | <0.001 |
| **SBP (mmHg)** | 118.97 ± 16.28 | 114.55 ± 14.97 | <0.001 |
| **DBP (mmHg)** | 74.00 ± 10.78 | 71.61 ± 10.50 | <0.001 |
| **FPG (mmol/L)** | 4.88 ± 0.56 | 5.16 ± 0.41 | <0.001 |
| **TC (mmol/L)** | 4.68 ± 0.90 | 5.13 ± 0.86 | <0.001 |
| **TG (mmol/L)** | 1.06 (0.72-1.60) | 0.73 (0.50-1.12) | <0.001 |
| **HDL-c (mmol/L)** | 1.37 ± 0.29 | 1.46 ± 0.40 | <0.001 |
| **LDL-c (mmol/L)** | 2.71 ± 0.67 | 3.09 ± 0.75 | <0.001 |
| **ALT(U/L)** | 18.00 (13.00-27.60) | 17.00 (13.00-23.00) | <0.001 |
| **AST(U/L)** | 22.00 (18.50-26.40) | 17.00 (14.00-21.00) | <0.001 |
| **AST/ALT** | 1.23 ± 0.45 | 1.04 ± 0.34 | <0.001 |
| **Gender, n (%)** |  |  | <0.001 |
| Female | 36356 (43.14%) | 6908 (45.18%) |  |
| Male | 47925 (56.86%) | 8383 (54.82%) |  |
| **Smoking, n (%)** |  |  | <0.001 |
| No | 66817 (79.28%) | 8904 (58.23%) |  |
| Yes | 17464 (20.72%) | 6387 (41.77%) |  |
| **Drinking, n (%)** |  |  | <0.001 |
| No | 69202 (82.11%) | 11661 (76.26%) |  |
| Yes | 15079 (17.89%) | 3630 (23.74%) |  |

Values are n (%), mean±SD or medians (quartiles)

BMI, body mass index; SBP, systolic blood pressure; DBP, diastolic blood pressure; FPG, fasting plasma glucose; TC, total cholesterol;TG, triglyceride; HDL-c, high-density lipoprotein cholesterol; LDL-c, low-density lipid cholesterol; AST, aspartate aminotransferase; ALT, alanine aminotransferase.

**Table S3. Relationship between AST/ALT and diabetes in different sensitivity analyses**

| **Exposure** | **Model I** | **Model II** |
| --- | --- | --- |
| **AST/ALT** | 0.549 (0.459, 0.657) <0.00001 | 0.537 (0.450, 0.640) <0.00001 |
| **AST/ALT (Quartile)** |  |  |
| **Q1** | Ref. | Ref. |
| **Q2** | 0.710 (0.617, 0.817) <0.00001 | 0.694 (0.605, 0.797) <0.00001 |
| **Q3** | 0.628 (0.532, 0.743) <0.00001 | 0.626 (0.532, 0.736) <0.00001 |
| **Q4** | 0.618 (0.505, 0.757) <0.00001 | 0.600 (0.492, 0.731) <0.00001 |
| **P for trend** | 0.832 (0.781, 0.886) <0.00001 | 0.826 (0.777, 0.878) <0.00001 |

Model I was sensitivity analysis in participants without CVD in the first two years of follow-up time (N=99511). We adjusted age, gender, BMI, SBP, DBP, FPG, LDL-c, HDL-c, TG, smoking and drinking status.

Model II was sensitivity analysis without adjustment for smoking and drinking status (N=99572). We adjusted age, gender, BMI, SBP, DBP, FPG, LDL-c, HDL-c, TG.

HR, Hazard ratios; CI: confidence, Ref: reference; AST, aspartate aminotransferase; ALT, alanine aminotransferase.
